# Prognostic value of the systemic immune-inflammation index in non-small cell lung cancer patients treated with immune checkpoint inhibitors: a systematic review and meta-analysis

**DOI:** 10.3389/fonc.2025.1532343

**Published:** 2025-05-16

**Authors:** Guorong Chen, Bahu Bao, Yucai Ye, Aoyan Hu, Jingzi Sun, Weiying Liu

**Affiliations:** ^1^ Department of Respiratory Medicine, The First Clinical Medical College of Lanzhou University, Lanzhou, China; ^2^ Department of Respiratory and Critical Care Medicine, the First Hospital of Lanzhou University, Lanzhou, Gansu, China

**Keywords:** systemic immune-inflammation index, non-small cell lung cancer, immune checkpoint inhibitors, survival, meta-analysis

## Abstract

**Background:**

Although the systemic immune-inflammation index (SII) has emerged as a potential prognostic marker in various cancers, its specific role in non-small cell lung cancer (NSCLC) patients undergoing immunotherapy remains insufficiently explored. To address this critical gap, we conducted a comprehensive meta-analysis to assess the prognostic value of SII in NSCLC patients treated with immune checkpoint inhibitors (ICIs).

**Method:**

A comprehensive search was conducted across multiple databases—including PubMed, EMBASE, Cochrane and Web of Science—to identify relevant studies. Hazard ratios (HRs) and 95% confidence intervals (CIs) were pooled to evaluate the prognostic significance of SII for survival outcomes.

**Result:**

Ten studies involving a total of 1,547 patients were included. High systemic immune-inflammation index (SII) was significantly associated with worse overall survival (OS) (HR=1.44, 95% CI=1.21–1.70, p < 0.001; I²=3.8%) and progression-free survival (PFS) (HR=1.44, 95% CI=1.21–1.71, p < 0.001; I²=37.2%). Subgroup analysis indicated that an SII >792 was significantly associated with poorer OS and PFS.

**Conclusion:**

High SII is significantly associated with poorer OS and PFS, particularly when SII >792.

**Systematic review registration:**

https://www.crd.york.ac.uk/PROSPERO/view/CRD42024586791, identifier RD42024586791.

## 1 Introduction

Lung cancer remains the most common and deadliest malignancy worldwide, with 2,480,301 new cases and 1,817,172 deaths reported in 2022 (1). NSCLC accounts for approximately 85% of these cases (2). The main treatment options include surgical resection, radiotherapy, chemotherapy, and targeted therapies. Immunotherapy has transformed cancer treatment, with ICIs showing promising results in NSCLC. These ICIs, particularly those targeting programmed cell death protein 1 (PD-1), programmed death-ligand 1 (PD-L1), and cytotoxic T-lymphocyte antigen 4 (CTLA-4), have significantly improved patient outcomes. However, despite these advancements, a significant challenge remains: over half of patients fail to respond to ICIs, even when combined with other therapies (3–6). This underscores the urgent need for reliable biomarkers to identify which patients are most likely to benefit from immunotherapy and to guide personalized treatment strategies. PD-L1 expression, a key biomarker for ICIs therapy, is routinely incorporated into clinical decision-making for NSCLC. However, reliance solely on PD-L1 expression has several limitations, reducing its predictive accuracy. First, some NSCLC patients with high PD-L1 expression (TPS >50%) fail to benefit from immunotherapy (7), whereas certain patients negative for PD-L1 do respond (8). Second, tumor types, intratumoral heterogeneity, and variations in detection methods may affect the accuracy and specificity of PD-L1 testing (9). Additionally, testing for PD-L1 and TMB requires tissue samples, making it a time-consuming and costly process (10). Therefore, there is an urgent need for more accessible and reliable biomarkers to improve patient selection and treatment outcomes. Emerging evidence highlights the significant role of inflammation and immune responses in tumor progression. Hematological markers such as the neutrophil-lymphocyte ratio (NLR) (11), platelet-lymphocyte ratio (PLR) (12), modified Glasgow Prognostic Score (mGPS) (13), and SII (14) have shown strong correlations with prognosis across various cancers. The SII, which combines NLR and PLR, is calculated by multiplying the platelet and neutrophil counts and dividing by the lymphocyte count. Initially proposed to predict outcomes in patients undergoing resection for hepatocellular carcinoma (14), SII has recently gained attention for its potential to predict survival in NSCLC patients treated with immunotherapy. While multiple studies suggest that high SII levels are associated with poorer survival outcomes in NSCLC patients undergoing immunotherapy (15–18), other research has not confirmed its prognostic value (19–24). This inconsistency highlights a critical gap in the understanding of SII’s role in patient prognosis. To address this uncertainty, this study performed a meta-analysis to examine the association between the systemic SII and prognosis in patients with advanced NSCLC treated with ICIs. The objective was to evaluate the prognostic value and reliability of SII as a biomarker for survival outcomes in patients with advanced NSCLC treated with ICIs.

## 2 Materials and methods

### 2.1 Search strategy

This study followed the Preferred Reporting Items for Systematic Reviews and Meta-Analyses (PRISMA) guidelines (25). The meta-analysis protocol was registered in the International Prospective Register of Systematic Reviews (PROSPERO) with the registration number CRD42024586791. Relevant studies published up to February 19, 2025, were systematically retrieved from PubMed, EMBASE, Cochrane and Web of Science databases without language restrictions. Both MeSH terms and free text keywords were used to maximize the sensitivity of the search. The primary search terms included, but were not limited to, the following: (“Systemic Immune Inflammation Index” OR “SII”)AND(”Non-Small-Cell Lung Carcinomas” OR “Non-Small Cell Lung Cancer” OR “NSCLC” )AND(”Immune Checkpoint Blockers” OR “Immune Checkpoint Inhibitor” OR “CTLA-4 Inhibitor” OR “PD-1 Inhibitor” OR “PD-L1 Inhibitor” OR “pembrolizumab” OR “Nivolumab” OR “sintilimab” OR “camrelizumab” OR “tislelizumab” OR “durvalumab” OR “atezolizumab” OR “sugemalimab” OR “lpilimumab” OR “tremelimumab”.)A detailed search strategy (taking PubMed as an example) was provided in the **Supplementary Material**. Additionally, the reference lists of relevant articles were manually reviewed to identify further eligible studies.

### 2.2 Inclusion and exclusion criteria

The inclusion criteria were as follows: (1) Patients with pathologically confirmed NSCLC. (2) Patients receiving treatment with ICIs. (3) The SII was calculated using the formula: (peripheral platelet count × neutrophil count)/lymphocyte count. (4) Studies that identified an optimal cutoff value for SII, dividing patients into high and low SII groups accordingly. (5) Studies evaluating the prognostic value of SII on survival outcomes, including OS or PFS, with HRs and 95%CIs explicitly reported in text or extractable from Kaplan-Meier survival curves. (6) Cohort studies, including both prospective and retrospective studies.

Exclusion criteria: (1) Patients with active infections, connective tissue diseases, hematologic disorders, or other autoimmune diseases were excluded. (2) Patients who underwent curative surgical resection were excluded. (3) Studies that did not clearly report the SII calculation method or the rationale for cutoff determination were excluded. (4) Studies lacking survival outcome data or from which such data could not be extracted were excluded. (5) Animal and cell-based experimental studies were excluded. (6) Letters, meta-analyses, editorials, expert opinions, case reports, and review articles were excluded. (7) In cases of overlapping study populations, the study with the largest sample size and most complete data was included in the meta-analysis.

### 2.3 Data extraction and quality assessment

Two investigators (G.C. and B.B.) independently assessed all studies, and any discrepancies were resolved through discussion with a third investigator (Y.Y.) until consensus was reached. The following data were extracted: first author’s name, publication year, country, sample size, study period, gender, age, smoking history, tumor histology, TNM stage, PD-L1 expression, treatment regimen, follow-up duration, SII cutoff value, cutoff determination method, survival outcomes, survival analysis method, and HRs with 95% CIs. When both multivariate and univariate analyses were performed, HRs and 95% CIs derived from the multivariate analysis were selected. Two independent authors (G.C. and B.B.) evaluated study quality using the Newcastle-Ottawa Scale (NOS), assessing selection of the study population (0-4 points), comparability of groups (0-2 points), and outcome measurement (0-3 points). NOS scores range from 0 to 9, with studies scoring >6 considered high quality. The details of the NOS scores are summarized in **Table 1**.

**Table 1:** The quality assessment scores by NOS of included studies.

Study	Year	Selection (0-4 points)				Comparability (0-2 points)	Outcome (0-3 points)			Total score
		Representativeness of the exposed cohort	Selection of the non exposed cohort	Ascertainment of exposure	Demonstration that outcome of interest was not present at start of study	Comparability of cohorts on the basis of the design or analysis	Assessment of outcome	Was follow-up long enough for outcomes to occur	Adequacy of follow up of cohorts	
Yamaguchi O (18)	2023	★	★	★	★	☆☆	★	☆	★	6
Seban RD (17)	2021	★	★	★	★	★☆	★	★	★	8
Liu J (21)	2019	★	★	★	★	☆☆	★	★	★	7
Holtzman L (22)	2022	★	★	★	★	★☆	★	★	★	8
Banna GL (15)	2022	★	★	★	★	★☆	★	★	★	8
Fang Q (19)	2023	★	★	★	★	★☆	★	★	☆	7
Rizzo A (22)	2023	★	★	★	★	★☆	★	★	★	8
Keiko T (23)	2023	★	★	★	★	★☆	★	☆	★	7
Arife Ulas (24)	2024	★	★	★	★	★☆	★	☆	☆	6
Safak YD (16)	2024	★	★	★	★	★☆	★	☆	☆	6

Newcastle-Ottawa Scale; a ★ represents 1 point; a ★ represents 0 point.

### 2.4 Statistical analysis

Combined HRs and 95% CIs were calculated to evaluate the prognostic value of SII for survival outcomes in NSCLC patients treated with immune checkpoint inhibitors. Heterogeneity among studies was assessed using the Q test and I²statistic. Fixed-effect models were applied when I²< 50% or the heterogeneity test P-value > 0.1; otherwise, random-effects models were used. I²value <25% indicates low heterogeneity, 25% < I²<50% suggests moderate heterogeneity, and I² >50% indicates substantial heterogeneity; I²value >75% is considered high heterogeneity. Heterogeneity analysis was conducted when I²exceeded 50%. We prespecified a series of subgroup analyses to assess the impact of different factors on the results. These subgroup analyses encompassed study design characteristics (e.g., sample size, survival analysis method, SII cutoff value and its determination method) as well as intervention details (treatment regimen and type of immune checkpoint inhibitors). In addition, we conducted a meta-regression analysis to explore the effects of continuous variables on our results, including sample size, median age, median follow-up duration, and NOS score. Publication bias was assessed visually using funnel plots, Begg’s test, and Egger’s test. All statistical analyses were performed using Stata 17.0 software (StataCorp LLC, TX, USA), with p < 0.05 considered statistically significant.

### 2.5 Ethics

As all data used in this meta-analysis were obtained from publicly available databases, approval from institutional review boards or ethics committees was not required.

## 3 Result

### 3.1 Search results

**Figure 1:**
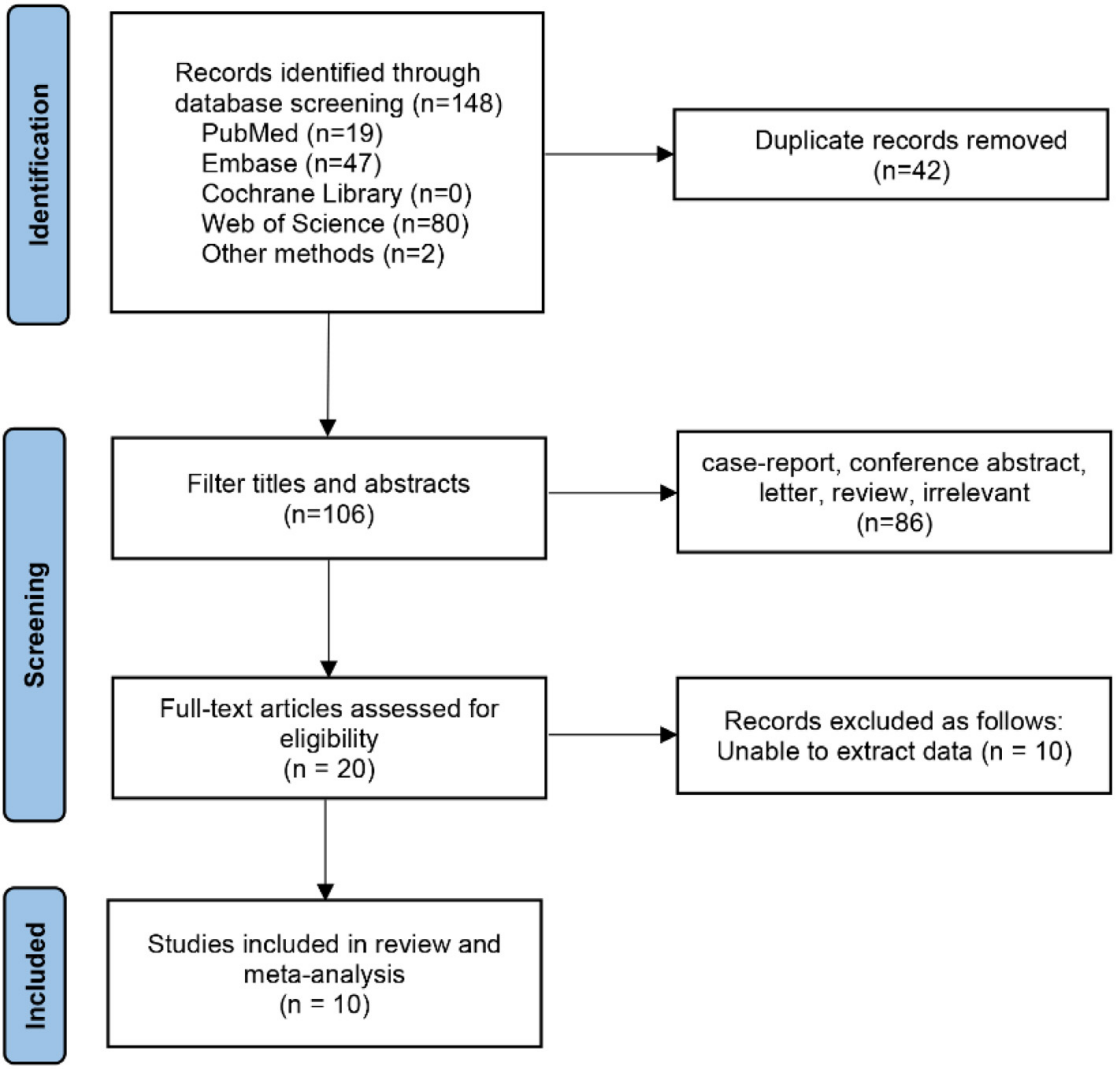
Flowchart of database search and study inclusion.

Initially, 148 relevant studies were identified, of which 10 studies involving 1,547 patients were finally included according to the inclusion and exclusion criteria (15–24). The literature screening process and results are shown in the **Figure 1**.

### 3.2 Baseline characteristics and study designs

Ten retrospective cohort studies published since 2019 were included, involving a total of 1,547 patients with NSCLC receiving immune checkpoint inhibitors from China (19, 21), Japan (18, 23), Turkey (16, 24), France (17), Israel (20), the United Kingdom (15), Switzerland (15), and Italy (22). One of the studies separately reported survival outcomes for patients receiving immune monotherapy and immune combination chemotherapy; therefore, we divided this study into two cohorts (Holtzman L 2022a and Holtzman L 2022b) for inclusion in the meta-analysis (20). In this study, patients from four cohorts received immunotherapy monotherapy (17, 18, 20, 21), five cohorts received immunotherapy combined with chemotherapy (15, 16, 19, 20, 23), and the remaining two cohorts used both approaches (22, 24). Regarding immune checkpoint inhibitors, Pembrolizumab was used in five cohorts (15, 17, 20, 22), Nivolumab in four cohorts (16, 18, 21, 24), and Durvalumab in one cohort (23). The sample sizes ranged from 43 to 308, with a median of 121 patients. Of these studies, five performed multivariate analyses (15–18, 20), five conducted univariate analyses (19, 20, 22–24), and one derived HRs and 95% CIs from Kaplan-Meier curves (21). The SII cutoff values ranged from 400 to 1,444 (median 792.07). Among these, five cohorts determined cutoff values using receiver operating characteristic (ROC) curves (16, 18, 21, 22, 24), three cohorts adopted cutoff values from previous literature (20, 23), two cohorts used median values (15, 19), and one cohort utilized X-tile software (17). The Newcastle-Ottawa Scale (NOS) scores of the included studies ranged from 6 to 8, indicating moderate to high methodological quality. The baseline characteristics and study designs of the included studies are summarized in the **Table 2** and **Table 3**.

**Table 2:** The baseline characteristics of the included studies.

Study	Country	Sample size	Study design	Study period	Sex (M/F)	Age, years median(range)	Smoking	Types of NSCLC	TNM stage	PD-L1 expression
Yamaguchi O (2023) (18)	Japan	101	Retrospective	2020.12-2022-03	83/18	72	Current/Former=91 Never=10	Squamous cell carcinoma=39 Adenocarcinoma = 54 Other=8	III=7 IV=94	< 1%=42 1–49%=48 ≥50%=11
Seban RD (2021) (17)	French	51	Retrospective	2016.11-2019.11	31/20	65 (37–86)	Current/Former=50 Never=1	Squamous cell carcinoma=12 Other=39	NA	< 1%=0 1–49%=51 ≥50%=51
Liu J (2019) (21)	China	44	Retrospective	2016.03-2018.07	33/11	60 (43‐74)	Current/Former=29 Never=15	Squamous cell carcinoma=13 Adenocarcinoma =31	IIIb=9 IV=35	NA
Holtzman L (2022) a (22)	Israel	302	Retrospective	2016.06-2020.12	200/102	70 (36-97)	Current/Former=268 Never=32 NA=2	Squamous cell carcinoma=52 Adenocarcinoma =227 Other=23	III=16 IV=284 NA=2	≥75%=115 <75%=73 NA=114
Holtzman L (2022) b (22)	Israel	121	Retrospective	2016.06-2020.12	74/47	66 (35-87)	Current/Former=108 Never=12 NA=1	Squamous cell carcinoma=77 Adenocarcinoma =29 Other=15	III=114 IV=7	≥75%=43 <75%=41 NA=37
Banna GL (2022) (15)	England+ Switzerland	308	Retrospective	2018.03-2021.04	171/137	65 (37–84)	Current/Former=283 Never=25	Squamous cell carcinoma=51 Adenocarcinoma =246 Other=11	IIIb=24 IV=284	Negative=165 Positive=111 High=20 NA=12
Fang Q (2023) (19)	China	223	Retrospective	2017.03-2019.03	189/34	Mean=60.4	NA	Squamous cell carcinoma=90 Adenocarcinoma =133	III=28 IV=195	Negative=45 Positive=73 NA=105
Rizzo A (2023) (22)	Italy	43	Retrospective	2018.10-2022.04	25/18	67 (61–72)	Current/Former=41 Never=2	Squamous cell carcinoma=8 Adenocarcinoma = 24 adenosquamous=1	IV=43	< 1%=5 1–49%=11 ≥50%=27
Keiko Tanimura (2023) (23)	Japan	126	Retrospective	2018.07-2021.03	98/28	71 (64.3-76)	Current/Former=16 Never=110	Squamous cell carcinoma=65 Adenocarcinoma = 57 Other=4	≤IIb= 11 IIIa= 48 IIIb= 56 IIIc=11	≥50%=41 <50%=50 NA=35
Arife Ulas (2024) (24)	Turkey	104	Retrospective	2019.10-2024.3	94/10	62 (39-82)	Current/Former=99 Never=5	Squamous cell carcinoma=48 Other=56	NA	Negative=48 Positive=24 NA=32
Safak Yildirim DISLI (2024) (16)	Turkey	124	Retrospective	2022.02-2023.06	110/14	Mean ± SD= 62.32 ± 8.43	Current/Former=108 Never=16	Squamous cell carcinoma=52 Adenocarcinoma = 64 Other=8	NA	NA

NSCLC, Non-Small Cell Lung Cancer; TNM, Tumor-Node-Metastasis; PD-L1, Programmed Cell Death-Ligand 1; PD-1, Programmed Cell Death Protein 1; NA, Not Available; SD, Standard Deviation.

**Table 3:** The study designs of the included studies.

Study	Treatment	ICIs	Follow-up(mouth) Median(range)	Cut-off value of SII	Cut-off determination	Survival outcomes	Survival analysis	NOS score
Yamaguchi O (2023) (18)	PD-1 inhibitor +CTLA4 antibody	Nivolumab+Ipilimumab	9.1	1,162.849	ROC analysis	OS; PFS	Multivariate	6
Seban RD (2021) (17)	PD-1 inhibitor	Pembrolizumab	26.5	1,207	Literature	OS; PFS	Multivariate	8
Liu J (2019) (21)	PD-1 inhibitor	Nivolumab	6.9 (0.6-28.5)	603.5	ROC analysis	OS; PFS	Kaplan-Meier	7
Holtzman L (2022) a (22)	PD-1 inhibitor	Pembrolizumab	28.6	400	Literature	OS	Multivariate	8
Holtzman L (2022) b (22)	PD-1 inhibitor+chemotherapy	Pembrolizumab	15.5	400	Literature	OS	Univariate	8
Banna GL (2022) (15)	PD-1 inhibitor+ platinum-based chemotherapy	Pembrolizumab	18.0 (15.9-20.1)	1,444	Median	OS; PFS	Multivariate	8
Fang Q (2023) (19)	PD-1 inhibitor+ chemotherapy	NA	20.4	792.07	Median	OS	Univariate	7
Rizzo A (2023) (22)	PD-1 inhibitor/PD-1 inhibitor+ chemotherapy	Pembrolizumab	18.2	1,235	ROC analysis	OS; PFS	Univariate	8
Keiko Tanimura (2023) (23)	CRT+PD-L1 inhibitor	Durvalumab	16.3	750	Literature	OS; PFS	Univariate	7
Arife Ulas (2024) (24)	PD-1 inhibitor/PD-1 inhibitor+ chemotherapy	Nivolumab	22	753.5	ROC analysis	OS; PFS	Univariate	6
Safak Yildirim DISLI (2024) (16)	CRT+PD-1 inhibitor	Nivolumab	18.86	1024.5	ROC analysis	OS	Multivariate	6

CTLA-4, Cytotoxic T Lymphocyte-Associated Antigen-4; ICIs, Immune Checkpoint Inhibitors; SII, Systemic Immune-Inflammation Index; NOS, Newcastle-Ottawa Scale; ROC, Receiver Operating Characteristic Curve; OS, Overall Survival; PFS, Progression-Free Survival; NA, Not Available.

### 3.3 Association between SII and overall survival in NSCLC immunotherapy

**Figure 2:**
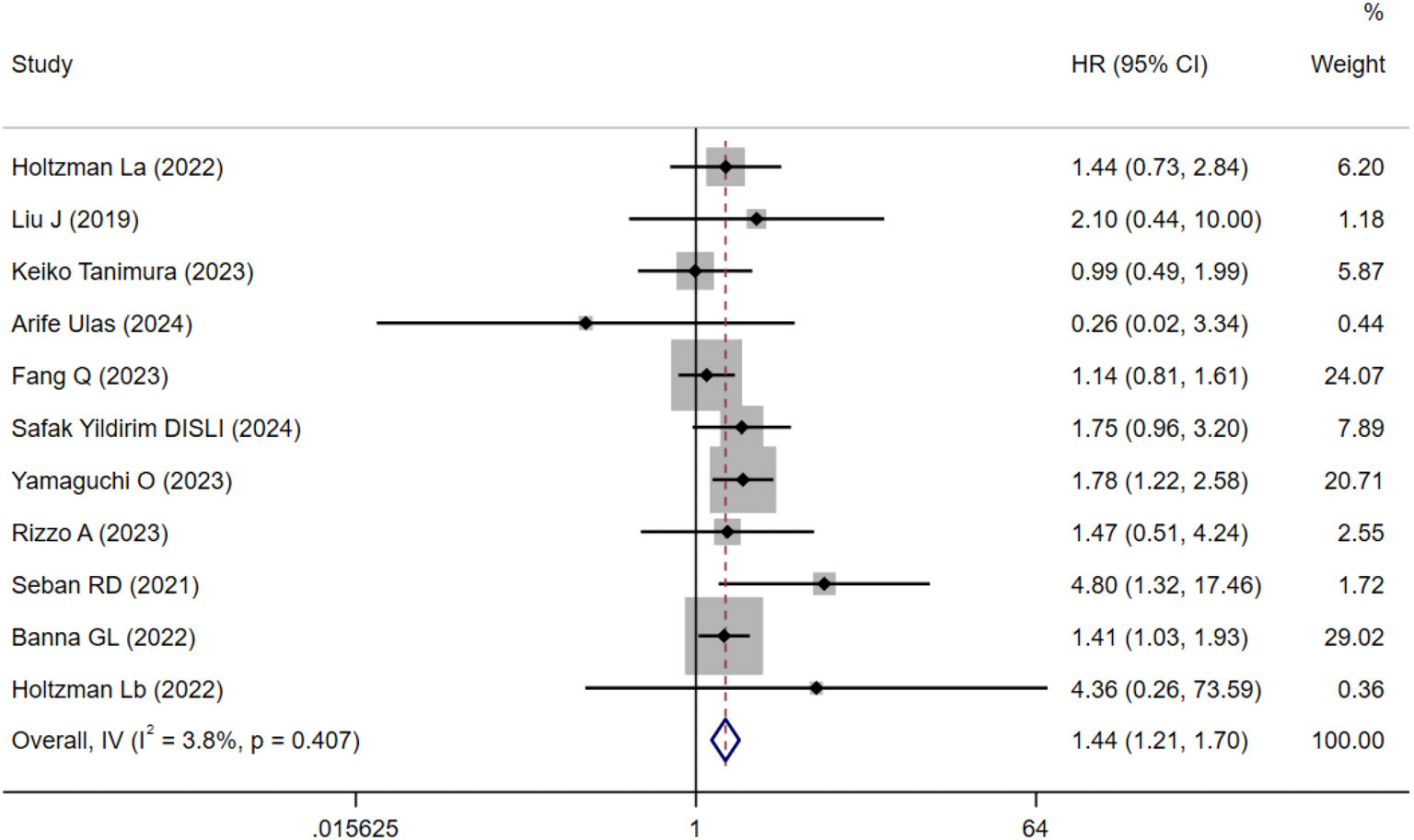
Forest plots for the meta-analysis of the association between SII and OS of NSCLC patients on ICIs.

A total of 11 cohort studies involving 1,547 patients reported the prognostic value of SII for OS in NSCLC patients treated with ICIs (15–24). Due to low heterogeneity (I²=3.8%, P = 0.407; **Figure 2**), a fixed-effects model was applied. Meta-analysis indicated that patients with high SII had significantly worse OS compared to those with low SII (HR=1.44, 95% CI=1.21–1.70, P < 0.001; **Figure 2**).

**Figure 3:**
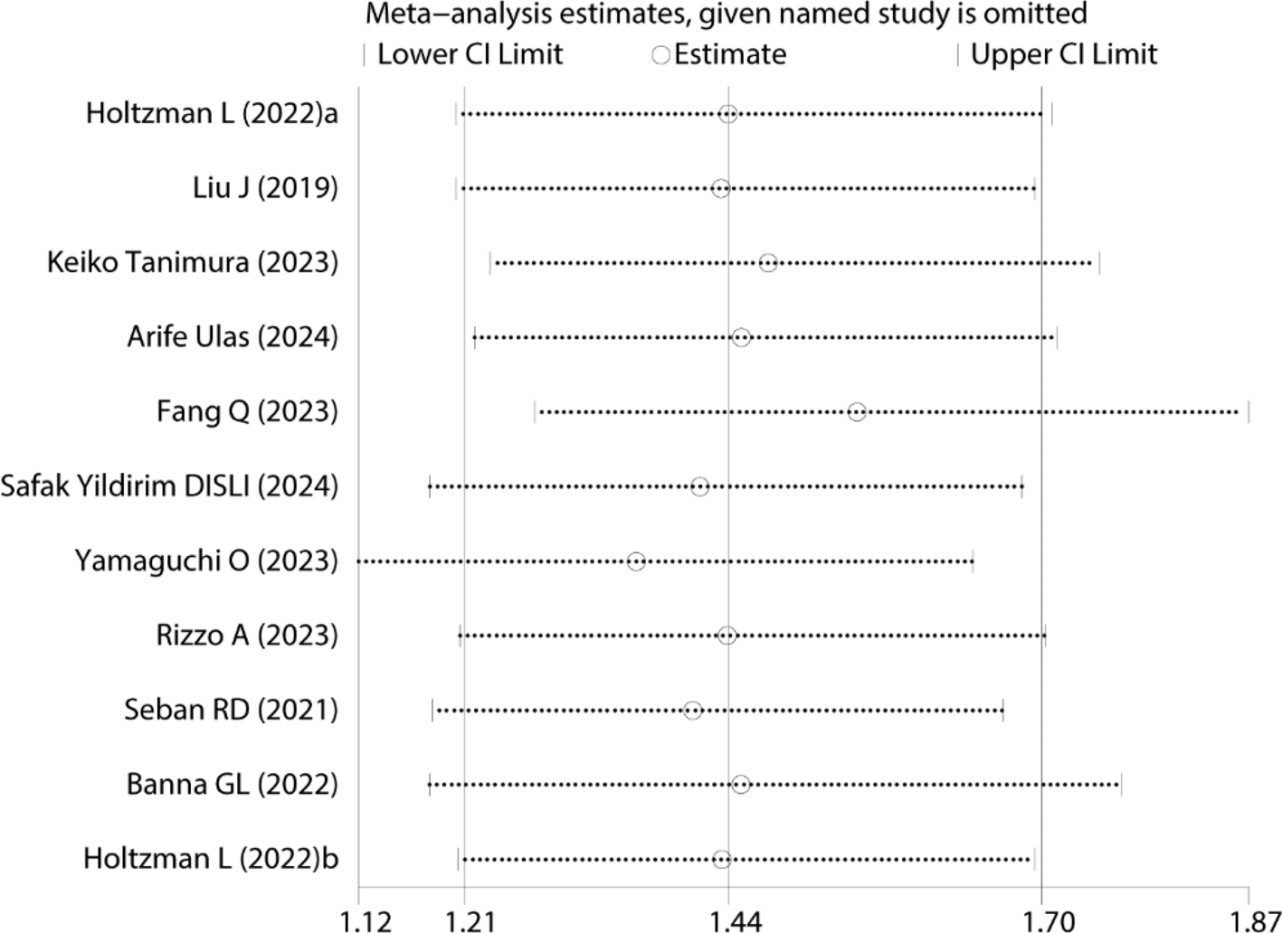
Sensitivity analyses of HRs and 95%CIs for OS.

Sensitivity analysis was performed to assess the robustness of the pooled OS results (**Figure 3**). The results remained statistically significant after sequentially excluding each study, indicating that the meta-analysis conclusions were not overly influenced by any single study, thus confirming their reliability and robustness.

Subgroup analyses showed all subgroups low heterogeneity and tests for differences in effect sizes between subgroups were not statistically significant (P>0.05; **Table 4**). This indicates that the prognostic value of SII for OS in NSCLC patients treated with ICIs remained stable, regardless of subgroup classification by sample size, survival analysis method, type of ICIs, or cutoff determination method. However, heterogeneity increased within the subgroup involving multiple immunotherapy regimens (I²=33.7%; **Table 4**). By comparing the key characteristics of the two studies in the original texts, we consider that this heterogeneity may result from differences in study designs related to treatment regimens, medications, drug doses, and hormonal pretreatment. Notably, SII >792.07 was significantly associated with poorer OS, whereas this association was markedly weaker in the subgroup with SII ≤792.07. This suggests that an SII value >792 may represent a potential threshold for identifying high-risk patients undergoing immunotherapy for NSCLC. Compared with univariate analysis (HR: 1.13; **Table 4**), multivariate analysis showed a more significant association with worse OS (HR: 1.61; **Table 4**). Similar trends were observed in immunotherapy monotherapy subgroups and those using ROC curves to determine cutoffs. This indicates that, after adjusting for confounding factors, the prognostic value of the ROC-derived SII cutoff for OS is more pronounced in NSCLC patients undergoing monotherapy with ICIs.

**Table 4:** Results of subgroup analysis for OS.

Variables	No. of studies	No. of patients	HR (95%CI)	P for interaction	Heterogeneity	
					I^2^(%)	Ph
Total	11	1547	1.44 (1.21-1.70)		3.8%	0.407
Sample size						
≤121	6	464	1.84 (1.33-2.54)	0.083	0%	0.420
>121	5	1083	1.31 (1.08-1.60)		0%	0.660
survival analysis						
Multivariate	5	886	1.61 (1.31-1.98)	0.136	0%	0.422
Univariate	5	617	1.13 (0.84-1.51)		0%	0.640
Kaplan-Meier	1	44	2.10 (0.44-10.0)		0%	<0.001
treatment						
ICIs	4	498	1.81 (1.33-2.47)	0.204	0%	0.445
ICIs+ Chemotherapy	5	902	1.31 (1.06-1.61)		0%	0.552
Mix	2	147	1.14 (0.43-3.03)		33.7%	0.219
PD-(L)1 agent						
Pembrolizumab	5	825	1.51 (1.16-1.97)	0.347	0%	0.430
Nivolumab	4	373	1.73 (1.27-2.36)		0%	0.533
Durvalumab	1	126	0.99 (0.49-1.99)		0%	<0.001
Cut-off value of SII						
≤792.07	6	920	1.18 (0.89-1.55)	0.070	0%	0.653
>792.07	5	627	1.62 (1.31-2.01)		0%	0.434
Cut-off determination						
ROC analysis	5	416	1.71 (1.27-2.30)	0.113	0%	0.685
Median	2	531	1.28 (1.02-1.61)		0%	0.372
Literature	3	549	1.25 (0.77-2.01)		0%	0.510
X-tile	1	51	4.80 (1.32-17.46)		0%	<0.001

SII, systemic immune-inflammation index; ROC, receiver operating characteristic curve; ICIs, immune checkpoint inhibitors; Mix, Therapeutic regimens incorporating immune checkpoint inhibitors.

Further meta-regression analysis demonstrated that the association between high SII and poor OS was not significantly influenced by study characteristics such as sample size, median age, median follow-up duration, or NOS score (P>0.05; **Table 5**).

**Table 5:** Results of univariate meta-regression analysis.

Variables	HR for OS		
	Coefficient	95% CI	P
Sample size	0.998	0.996-1.001	0.281
age (years)	1.020	0.975-1.067	0.338
Follow-up duration (months)	0.987	0.950-1.026	0.474
NOS	0.961	0.735-1.258	0.751

SII, systemic immune-inflammation index; OS, overall survival; HR, hazard ratio; CI, confidence interval; NOS, Newcastle-Ottawa Scale.

### 3.4 Association between SII and progression-free survival in NSCLC immunotherapy

**Figure 4:**
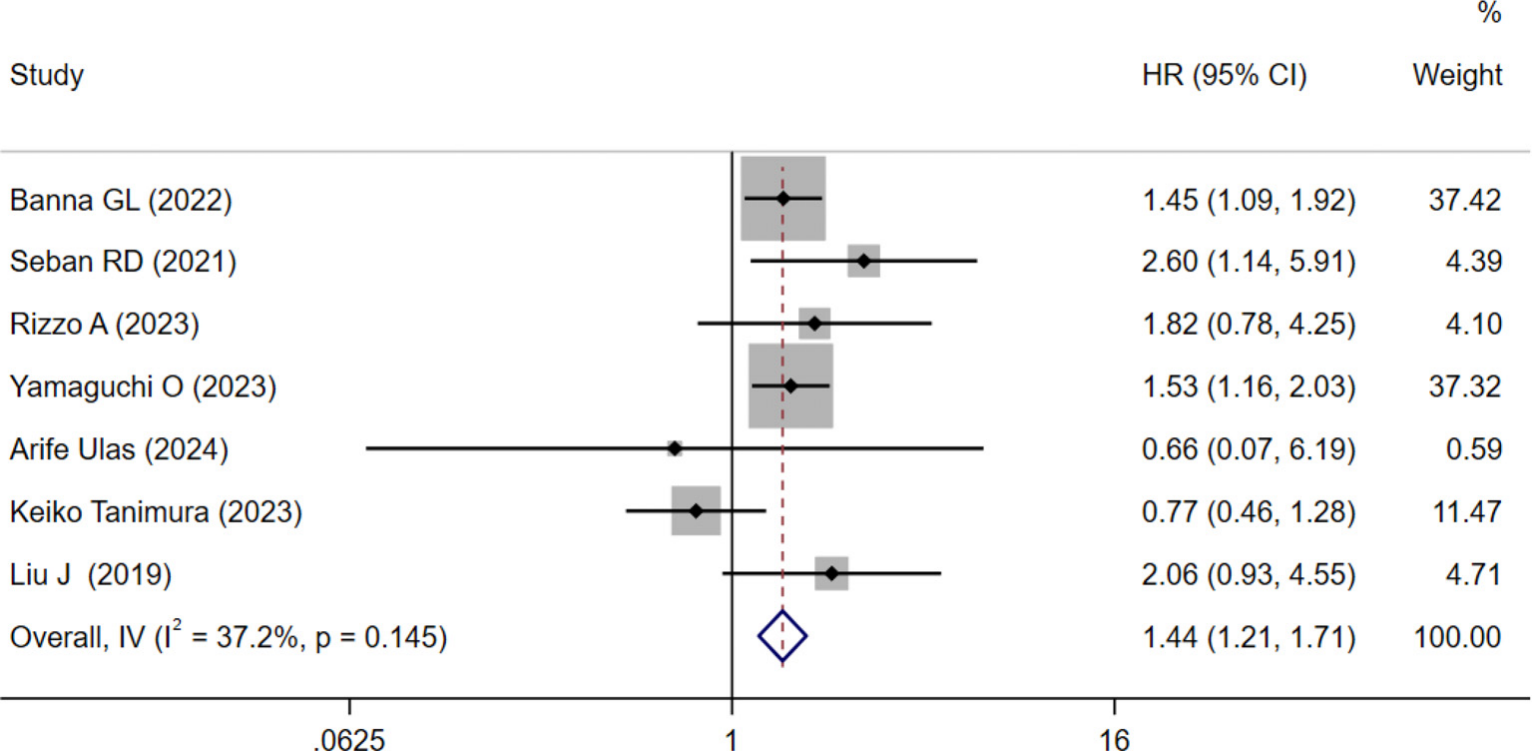
Forest plots for the meta-analysis of the association between SII and PFS of NSCLC patients on ICIs.

Seven cohorts involving 777 patients explored the prognostic value of SII for PFS in NSCLC patients treated with ICIs (15, 17, 18, 21–24). The results indicated moderate heterogeneity (I² = 37.2%, P = 0.145; **Figure 4**). The pooled HR was 1.44 (95% CI: 1.21–1.71, p < 0.001; **Figure 4**), indicating that high SII was associated with worse PFS.

**Figure 5:**
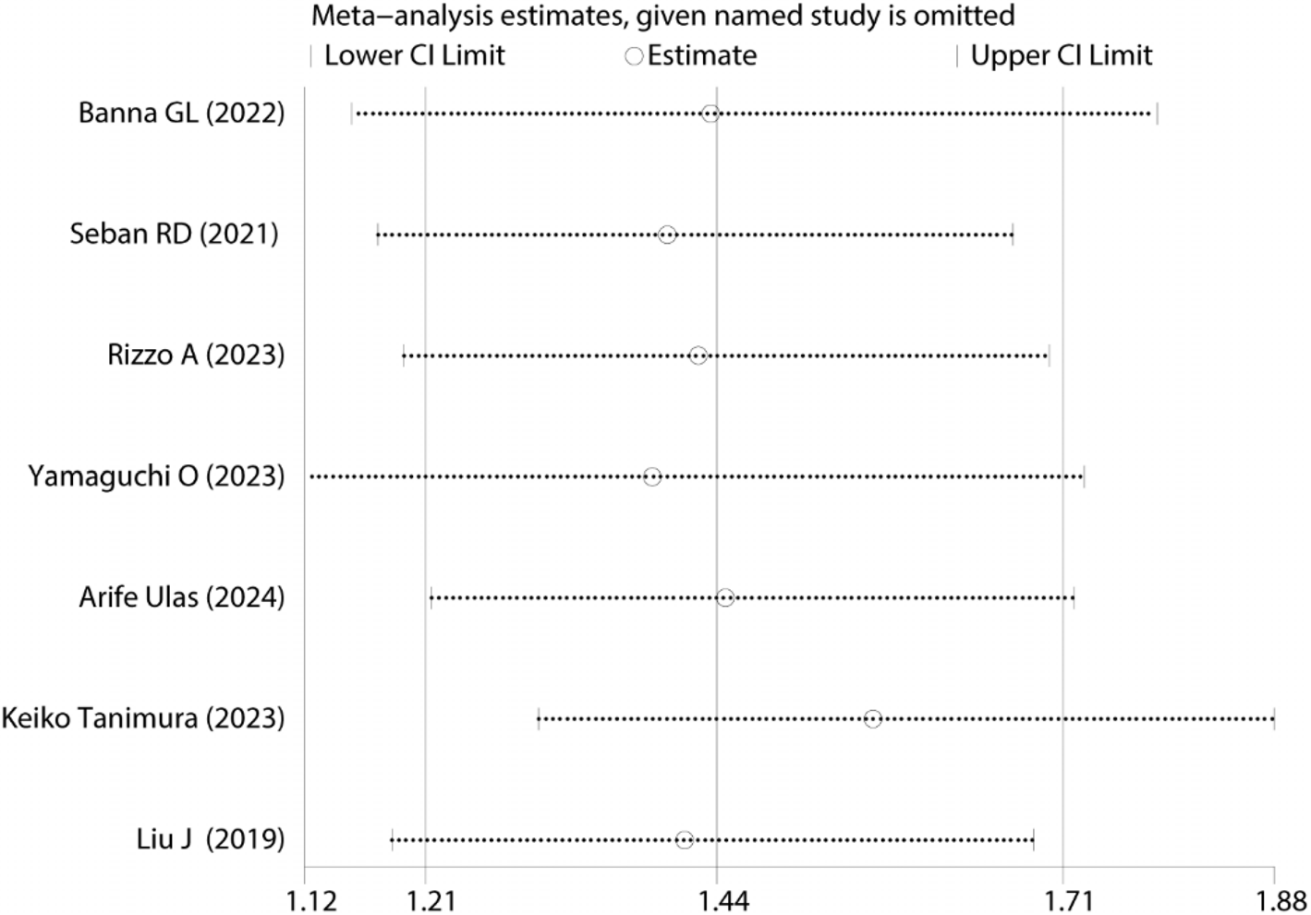
Sensitivity analyses of HRs and 95%CIs for PFS.

Sensitivity analysis was conducted to evaluate the robustness of pooled PFS results **Figure 5**. The results remained statistically significant upon sequential exclusion of individual studies, demonstrating that no single study disproportionately influenced the original meta-analysis conclusions, thus confirming the reliability and robustness of the findings.

Subgroup analysis showed reduced heterogeneity in groups categorized by different types of ICIs and cutoff determination methods, the test for differences in effect sizes between subgroups was statistically significant (P < 0.05; **Table 6**), suggesting these factors as potential sources of heterogeneity. Detailed heterogeneity analysis was conducted for three subgroups with substantial heterogeneity (sample size >121, immunotherapy combined with chemotherapy, and cutoff value ≤792.07), considering several potential influencing factors, including but not limited to: 1. Medications: The influence of different drugs and variations among manufacturers of the same drug cannot be ruled out. However, due to the lack of detailed descriptions in included studies, further analysis was not feasible. 2. Cutoff determination methods: Different approaches to defining cutoff values may impact final outcomes. However, studies included in this analysis were of high quality, with reliable statistical methodologies, making results meaningful. Additionally, SII values >792.07 were significantly associated with worse PFS; however, this association was notably weaker in the subgroup with SII ≤792.07. This suggests that an SII value >792 may serve as a potential predictive threshold for poorer PFS in NSCLC patients receiving ICIs. The subgroup treated with immunotherapy monotherapy and the subgroup using ROC curve analysis to determine optimal cutoff values showed stronger associations with poorer PFS compared to other subgroups based on identical classification criteria. The results demonstrated that in immunotherapy monotherapy settings, SII cutoff values determined by ROC curve analysis showed enhanced prognostic predictive utility for PFS in NSCLC patients receiving ICIs.

**Table 6:** Results of subgroup analysis for PFS.

Variables	No. of studies	No. of patients	HR (95%CI)	P for interaction	Heterogeneity	
					I^2^(%)	Ph
Total	7	777	1.44 (1.21-1.71)		37.2%	0.145
Sample size						
≤121	3	138	2.14 (1.33-3.44)	0.079	0%	0.833
>121	4	639	1.36 (1.13-1.63)		50.8%	0.107
survival analysis						
Multivariate	3	460	1.54 (1.27-1.86)	0.091	0%	0.419
Univariate	3	273	0.95 (0.62-1.46)		33.6%	0.222
Kaplan-Meier	1	44	2.06 (0.93-4.55)		0%	<0.001
treatment						
ICIs	3	196	1.66 (1.29-2.13)	0.280	0%	0.417
ICIs+ Chemotherapy	2	434	1.25 (0.98-1.60)		78.1%	0.033
Mix	2	147	1.60 (0.72-3.54)		0%	0.406
PD-(L)1 agent						
Pembrolizumab	3	402	1.56 (1.21-2.02)	0.036	0%	0.392
Nivolumab	3	249	1.56 (1.20-2.03)		0%	0.590
Durvalumab	1	126	0.77 (0.46-1.28)		0%	<0.001
Cut-off value of SII						
≤792.07	3	274	1.01 (0.66-1.54)	0.068	53.9%	0.114
>792.07	4	503	1.55 (1.28-1.87)		0%	0.597
Cut-off determination						
ROC analysis	4	292	1.58 (1.23-2.04)	0.039	0%	0.760
Median	1	308	1.45 (1.09-1.92)		0%	<0.001
Literature	1	126	0.77 (0.46-1.28)		0%	<0.001
X-tile	1	51	2.60 (1.14-5.91)		0%	<0.001

SII, systemic immune-inflammation index; ROC, receiver operating characteristic curve; ICIs, immune checkpoint inhibitors; Mix, Therapeutic regimens incorporating immune checkpoint inhibitors.

Given the limited number of included studies (n = 7), which did not meet the conventional requirement for meta-regression analysis (generally ≥10 studies for statistical power), meta-regression was not performed.

### 3.5 Publication bias Begg’s funnel plots

**Figure 6:**
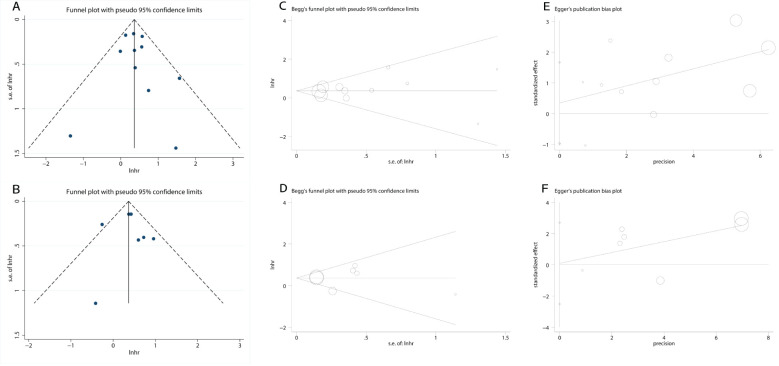
Plots for publication bias test. **(A)** Funnel plot for OS; **(B)** Funnel plot for PFS; **(C)** Begg’s test for OS; P=0.533; **(D)** Begg’s test for PFS; P=0.764; **(E)** Egger’s test for OS; P=0.566; **(F)** Egger’s test for PFS; P=0.903.

Funnel plots were generated to assess publication bias. The symmetry test (**Figure 6**) revealed a generally symmetrical distribution, suggesting no substantial publication bias across studies. Additionally, Begg’s and Egger’s tests were further employed for quantitative evaluation of publication bias. Results showed nonsignificant bias: OS group (Begg’s P = 0.533; Egger’s P = 0.566; **Figure 6**), PFS group (Begg’s P = 0.764; Egger’s P = 0.903; **Figure 6**). All P values > 0.05, indicating no detectable publication bias in the included studies.

## 4 Discussion

While PD-L1 expression assays are essential for selecting patients for immunotherapy, their limitations highlight the need for additional biomarkers to better identify those most likely to benefit. In this context, a study investigating the association between PD-L1 expression and peripheral blood inflammatory parameters in patients with NSCLC demonstrated a statistically significant difference in SII between patients with PD-L1 TPS≥50% and those with TPS <50%. Notably, patients with high PD-L1 expression (TPS≥50%) had significantly lower SII levels compared with those having low PD-L1 expression (TPS <50%). Further analysis revealed a significant negative correlation between SII and PD-L1 TPS expression levels (26). For example, a meta-analysis by Yan Wang et al. encompassing 17 studies found that a higher SII was significantly associated with poorer OS and PFS in cancer patients treated with ICIs (27). Similarly, Junyan Kou and colleagues conducted a meta-analysis of 2,438 patients with advanced cancers, demonstrating that high SII correlated with lower objective response rates, reduced disease control rates, and shorter OS and PFS (28).

This meta-analysis, which included 11 cohorts comprising 1,547 patients, revealed that higher SII was significantly associated with poorer OS and PFS in patients with NSCLC treated with ICIs. Notably, SII values greater than 792.07 showed a statistically significant association with survival outcomes. This indicates that an SII cutoff value of 792 may represent a critical threshold for risk stratification and therapeutic decision-making. Additionally, multivariable Cox regression analysis, immunotherapy monotherapy cohorts, and ROC curve-optimized SII cutoff values significantly enhanced the predictive performance of SII for OS and PFS in NSCLC patients receiving ICIs. Specifically, multivariable survival analysis accounts for potential confounding factors, allowing for a more accurate assessment of the true impact of SII on patient prognosis. The SII cutoff values determined by ROC curve analysis enable more precise risk stratification, thereby improving the prediction of OS and PFS. In the immunotherapy monotherapy setting, where treatment variables are relatively consistent, SII demonstrates a stronger predictive effect on prognosis. In contrast, in combination therapy regimens, cytotoxic agents may exert myelosuppressive effects, such as altering peripheral blood cell counts, which could obscure the independent prognostic impact of inflammatory responses. Therefore, monotherapy better reflects the immune microenvironment imbalance indicated by SII.

The SII is calculated using platelet, neutrophil, and lymphocyte counts, any variation in the counts of these three cell types could affect the final value of SII. An increase in platelets and neutrophils or a decrease in lymphocytes leads to a higher SII, which is often associated with a more significant tumor burden, metastasis, and enhanced immunosuppression. Tumor cells activate platelets, causing them to form microaggregates that help the tumor evade immune surveillance. These platelets release cytokines like interleukin-6 (IL-6) and platelet-derived growth factor (PDGF), which further promote platelet production. By secreting growth factors and facilitating angiogenesis and metastasis, platelets contribute to tumor progression and invasion (29, 30). Simultaneously, the inflammatory tumor microenvironment and metastasis increase the secretion of neutrophil-stimulating factors such as granulocyte colony-stimulating factor (G-CSF), leading to elevated neutrophil levels. Neutrophils promote tumor initiation and proliferation by releasing pro-inflammatory molecules like miR-23a, miR-155, and reactive oxygen species (ROS). They also support angiogenesis by secreting vascular endothelial growth factor (VEGF) and matrix metalloproteinase 9 (MMP-9), further driving tumor progression (31). In contrast, immunosuppressive factors in the tumor microenvironment—such as transforming growth factor-beta (TGF-β) and PD-L1—impair the anti-tumor functions of lymphocytes, leading to a reduction in their numbers (32). ICIs enhance anti-tumor immune responses by blocking these immune checkpoints, which not only restores T-cell function but also inhibits the pro-tumor activity of neutrophils (33). Higher SII reflects the inflammatory and immune dysregulation status of cancer patients, correlating not only with poorer prognosis in NSCLC but also potentially predicting a reduced response to ICI treatment. In contrast, the SII values in healthy individuals are typically lower. A recent large-scale epidemiological study provided a reference range for SII based on nearly 30,000 healthy Chinese adults, reporting a 2.5–97.5 percentile reference interval of approximately 162–811 for males and 165–792 for females (34). This finding suggests that most healthy individuals have SII values below 800, which further supports our observation that NSCLC patients with an SII above 792 exhibit significantly poorer OS and PFS after treatment with immune checkpoint inhibitors.

As a potential prognostic biomarker, SII offers advantages over PD-L1 expression, including ease of sample collection, safety, reproducibility, standardization, low cost, and rapid result availability, thus showing great promise for timely clinical decision-making. During treatment, periodic monitoring of SII may help evaluate prognosis and therapeutic response, guiding interventions such as inflammation control, lymphocyte proliferation and activation, and antiplatelet therapy in patients with elevated SII levels. Additionally, combining SII with other biomarkers or clinical indicators could optimize clinical decision-making and assist in selecting the optimal therapeutic strategy for patients.

Potential limitations of this meta-analysis: 1. Subgroup analysis indicated that different ICIs did not significantly impact the predictive performance of SII. However, only ten studies met the inclusion criteria, resulting in a limited number of studies within subgroups and potentially insufficient statistical power. Furthermore, inter-study variability in treatment protocols – including therapeutic agents, manufacturers, dosages, pretreatment regimens, and treatment durations – potentially introduced confounding effects on outcome assessments. But these parameters were insufficiently detailed in original reports. Therefore, future studies should conduct large-scale, multicenter prospective trials. Moreover, strict control over these variables should be ensured during the study design phase to minimize confounding effects on outcome assessment. 2. The included studies excluded confounding factors such as acute infections, connective tissue diseases, and hematological disorders, ensuring the stability of SII. However, most studies did not provide detailed descriptions of patients’ underlying conditions (e.g., hypertension, diabetes) or long-term medication history, which may have led to insufficient control of potential confounders. Future research should improve data collection on comorbidities and long-term medication use to allow for better adjustment of confounding variables in multivariate analysis. 3. Differences in cutoff determination methods directly influence the SII cutoff values, contributing to increased heterogeneity among studies and potentially affecting the accuracy of SII predictions. Future studies should standardize the approach for determining cutoff values (e.g., ROC curve analysis) to ensure accurate risk stratification and better control for confounding factors.

## 5 Conclusion

This meta-analysis systematically evaluated the prognostic value of SII in NSCLC patients receiving ICIs. The results demonstrated that high SII was significantly associated with poorer OS and PFS, particularly when SII exceeded 792. These findings suggest that SII may serve as a prognostic biomarker for immunotherapy in NSCLC patients, aiding in the identification of individuals more likely to benefit from ICIs treatment. Further large-scale, multicenter prospective studies are warranted to validate the prognostic significance of SII in NSCLC patients receiving ICIs therapy.

## Data Availability

The original contributions presented in the study are included in the article/**Supplementary Material**. Further inquiries can be directed to the corresponding author.

## References

[1] Bray F Laversanne M Sung H Ferlay J Siegel RL Soerjomataram I . Global cancer statistics 2022: GLOBOCAN estimates of incidence and mortality worldwide for 36 cancers in 185 countries. CA: A Cancer J clinic. (2024) 74:229–63. doi: 10.3322/caac.21834 38572751

[2] Travis WD Brambilla E Riely GJ . New pathologic classification of lung cancer: relevance for clinical practice and clinical trials. J Clin Oncol. (2013) 31:992–1001. doi: 10.1200/JCO.2012.46.9270 23401443

[3] Rodríguez-Abreu D Powell SF Hochmair MJ Gadgeel S Esteban E Felip E . Pemetrexed plus platinum with or without pembrolizumab in patients with previously untreated metastatic nonsquamous NSCLC: protocol-specified final analysis from KEYNOTE-189. Ann Oncol. (2021) 32:881–95. doi: 10.1016/j.annonc.2021.04.008 33894335

[4] Socinski MA Jotte RM Cappuzzo F Nishio M Mok TSK Reck M . Association of immune-related adverse events with efficacy of atezolizumab in patients with non-small cell lung cancer: pooled analyses of the phase 3 IMpower130, IMpower132, and IMpower150 randomized clinical trials. JAMA Oncol. (2023) 9:527–35. doi: 10.1001/jamaoncol.2022.7711 PMC9936386 36795388

[5] Mok TSK Wu YL Kudaba I Kowalski DM Cho BC Turna HZ . Pembrolizumab versus chemotherapy for previously untreated, PD-L1-expressing, locally advanced or metastatic non-small-cell lung cancer (KEYNOTE-042): a randomised, open-label, controlled, phase 3 trial. Lancet (London England). (2019) 393:1819–30. doi: 10.1016/S0140-6736(18)32409-7 30955977

[6] Reck M Rodríguez-Abreu D Robinson AG Hui R Csőszi T Fülöp A . Pembrolizumab versus chemotherapy for PD-L1-positive non-small-cell lung cancer. New Engl J Med. (2016) 375:1823–33. doi: 10.1056/NEJMoa1606774 27718847

[7] de Castro G Jr. Kudaba I Wu YL Lopes G Kowalski DM Turna HZ . Five-year outcomes with pembrolizumab versus chemotherapy as first-line therapy in patients with non-small-cell lung cancer and programmed death ligand-1 tumor proportion score ≥ 1% in the KEYNOTE-042 study. J Clin Oncol. (2023) 41:1986–91. doi: 10.1200/jco.21.02885 PMC10082298 36306479

[8] Brahmer JR Lee JS Ciuleanu TE Bernabe Caro R Nishio M Urban L . Five-year survival outcomes with nivolumab plus ipilimumab versus chemotherapy as first-line treatment for metastatic non-small-cell lung cancer in checkMate 227. J Clin Oncol. (2023) 41:1200–12. doi: 10.1200/jco.22.01503 PMC9937094 36223558

[9] Rother C John T Wong A . Biomarkers for immunotherapy resistance in non-small cell lung cancer. Front Oncol. (2024) 14:1489977. doi: 10.3389/fonc.2024.1489977 39749035 PMC11693593

[10] Sholl LM . Biomarkers of response to checkpoint inhibitors beyond PD-L1 in lung cancer. Modern Pathol. (2022) 35:66–74. doi: 10.1038/s41379-021-00932-5 34608245

[11] Capone M Giannarelli D Mallardo D Madonna G Festino L Grimaldi AM . Baseline neutrophil-to-lymphocyte ratio (NLR) and derived NLR could predict overall survival in patients with advanced melanoma treated with nivolumab. J immunother Cancer. (2018) 6:74. doi: 10.1186/s40425-018-0383-1 30012216 PMC6048712

[12] Yang D Li P Meng Z Hu X Huang Z Huang H . Combined pretreatment neutrophil-lymphocyte ratio and platelet-lymphocyte ratio predicts survival and prognosis in patients with non-metastatic nasopharyngeal carcinoma: a retrospective study. Sci Rep. (2024) 14:9898. doi: 10.1038/s41598-024-59131-2 38688967 PMC11061272

[13] Brown JT Liu Y Shabto JM Martini D Ravindranathan D Hitron EE . Modified Glasgow Prognostic Score associated with survival in metastatic renal cell carcinoma treated with immune checkpoint inhibitors. J immunother Cancer. (2021) 9. doi: 10.1136/jitc-2021-002851 PMC8323383 34326170

[14] Hu B Yang XR Xu Y Sun YF Sun C Guo W . Systemic immune-inflammation index predicts prognosis of patients after curative resection for hepatocellular carcinoma. Clin Cancer Res. (2014) 20:6212–22. doi: 10.1158/1078-0432.CCR-14-0442 25271081

[15] Banna GL Cantale O Muthuramalingam S Cave J Comins C Cortellini A . Efficacy outcomes and prognostic factors from real-world patients with advanced non-small-cell lung cancer treated with first-line chemoimmunotherapy: The Spinnaker retrospective study. Int Immunopharm. (2022) 110:108985. doi: 10.1016/j.intimp.2022.108985 35777264

[16] Disli SY Ayas E Disli AK Ozdemir F . Prognostic value of inflammatory and nutritional index in advanced stage non-small cell lung cancer patients treated with nivolumab in second-line therapy. Uhod-Uluslararasi Hematoloji-Onkoloji Dergisi. (2024) 34:68–73. doi: 10.4999/uhod.247680

[17] Seban RD Assié JB Giroux-Leprieur E Massiani MA Bonardel G Chouaid C . Prognostic value of inflammatory response biomarkers using peripheral blood and [18F]-FDG PET/CT in advanced NSCLC patients treated with first-line chemo- or immunotherapy. Lung Cancer. (2021) 159:45–55. doi: 10.1016/j.lungcan.2021.06.024 34311344

[18] Yamaguchi O Kaira K Imai H Mouri A Shiono A Miura Y . Clinical utility of inflammatory and nutritious index as therapeutic prediction of nivolumab plus ipilimumab in advanced NSCLC. Oncology. (2024) 102:271–82. doi: 10.1159/000534169 37725914

[19] Fang Q Yu J Li W Luo J Deng Q Chen B . Prognostic value of inflammatory and nutritional indexes among advanced NSCLC patients receiving PD-1 inhibitor therapy. Clin Exp Pharmacol Physiol. (2023) 50:178–90. doi: 10.1111/1440-1681.13740 PMC10107359 36419356

[20] Holtzman L Moskovitz M Urban D Nechushtan H Keren S Reinhorn D . dNLR-based score predicting overall survival benefit for the addition of platinum-based chemotherapy to pembrolizumab in advanced NSCLC with PD-L1 tumor proportion score ≥50%. Clin Lung Cancer. (2022) 23:122–34. doi: 10.1016/j.cllc.2021.12.006 35034862

[21] Liu J Li S Zhang S Liu Y Ma L Zhu J . Systemic immune-inflammation index, neutrophil-to-lymphocyte ratio, platelet-to-lymphocyte ratio can predict clinical outcomes in patients with metastatic non-small-cell lung cancer treated with nivolumab. J Clin Lab Anal. (2019) 33:e22964. doi: 10.1002/jcla.22964 31282096 PMC6805305

[22] Rizzo A Cantale O Mogavero A Garetto L Racca M Venesio T . Assessing the role of colonic and other anatomical sites uptake by [(18) F]FDG-PET/CT and immune-inflammatory peripheral blood indexes in patients with advanced non-small cell lung cancer treated with first-line immune checkpoint inhibitors. Thorac Cancer. (2023) 14:2473–83. doi: 10.1111/1759-7714.15032 PMC10447168 37442801

[23] Tanimura K Takeda T Yoshimura A Honda R Goda S Shiotsu S . Predictive Value of Modified Glasgow Prognostic Score and Persistent Inflammation among Patients with Non-Small Cell Lung Cancer Treated with Durvalumab Consolidation after Chemoradiotherapy: A Multicenter Retrospective Study. Cancers. (2023) 15. doi: 10.3390/cancers15174358 PMC10486354 37686634

[24] Ulas A Temel B Kos FT . Comparison of prognostic values of seven immune indexes in advanced non-small-cell lung cancer treated with nivolumab: how effective can they be regarding our treatment decisions? Med (Kaunas). (2024) 60. doi: 10.3390/medicina60111792 PMC11596302 39596977

[25] Moher D Liberati A Tetzlaff J Altman DG . Preferred reporting items for systematic reviews and meta-analyses: the PRISMA statement. J Clin Epidemiol. (2009) 62:1006–12. doi: 10.1016/j.jclinepi.2009.06.005 19631508

[26] Bilgin B Hızal M Yücel Ş Şendur MAN Akyürek N Akıncı MB . The association of clinicopathologic features and peripheral blood parameters with high PD-L1 expression in non-small cell lung cancer. Tuberkuloz ve toraks. (2020) 68:118–25. doi: 10.5578/tt.69182 32755111

[27] Wang Y Ni Q . Prognostic and clinicopathological significance of Systemic Immune-Inflammation Index in cancer patients receiving immune checkpoint inhibitors: a meta-analysis. Ann Med. (2023) 55:808–19. doi: 10.1080/07853890.2023.2181983 PMC10795596 36892953

[28] Kou J Huang J Li J Wu Z Ni L . Systemic immune-inflammation index predicts prognosis and responsiveness to immunotherapy in cancer patients: a systematic review and meta−analysis. Clin Exp Med. (2023) 23:3895–905. doi: 10.1007/s10238-023-01035-y 36966477

[29] Li S Lu Z Wu S Chu T Li B Qi F . The dynamic role of platelets in cancer progression and their therapeutic implications. Nat Rev Cancer. (2024) 24:72–87. doi: 10.1038/s41568-023-00639-6 38040850

[30] Roweth HG Battinelli EM . Lessons to learn from tumor-educated platelets. Blood. (2021) 137:3174–80. doi: 10.1182/blood.2019003976 PMC8351883 33940594

[31] Huang X Nepovimova E Adam V Sivak L Heger Z Valko M . Neutrophils in Cancer immunotherapy: friends or foes? Mol Cancer. (2024) 23:107. doi: 10.1186/s12943-024-02004-z 38760815 PMC11102125

[32] Kishton RJ Sukumar M Restifo NP . Metabolic regulation of T cell longevity and function in tumor immunotherapy. Cell Metab. (2017) 26:94–109. doi: 10.1016/j.cmet.2017.06.016 28683298 PMC5543711

[33] Yi M Li T Niu M Mei Q Zhao B Chu Q . Exploiting innate immunity for cancer immunotherapy. Mol Cancer. (2023) 22:187. doi: 10.1186/s12943-023-01885-w 38008741 PMC10680233

[34] Liu Q Xu A Hang H Chen X Dai Y Wang M . Establishment of reference intervals for SII, NLR, PLR, and LMR in healthy adults in Jiangsu region in eastern China. Clin Lab. (2023) 69. doi: 10.7754/Clin.Lab.2022.220837 37145057

