# Supplementary figures and images for: Prognostic value of the systemic immune-inflammation index in non-small cell lung cancer patients treated with immune checkpoint inhibitors: a systematic review and meta-analysis
Source: Front Oncol. 2025 May 16;15:1532343. doi: 10.3389/fonc.2025.1532343 (PMC12122344; doi:10.3389/fonc.2025.1532343)

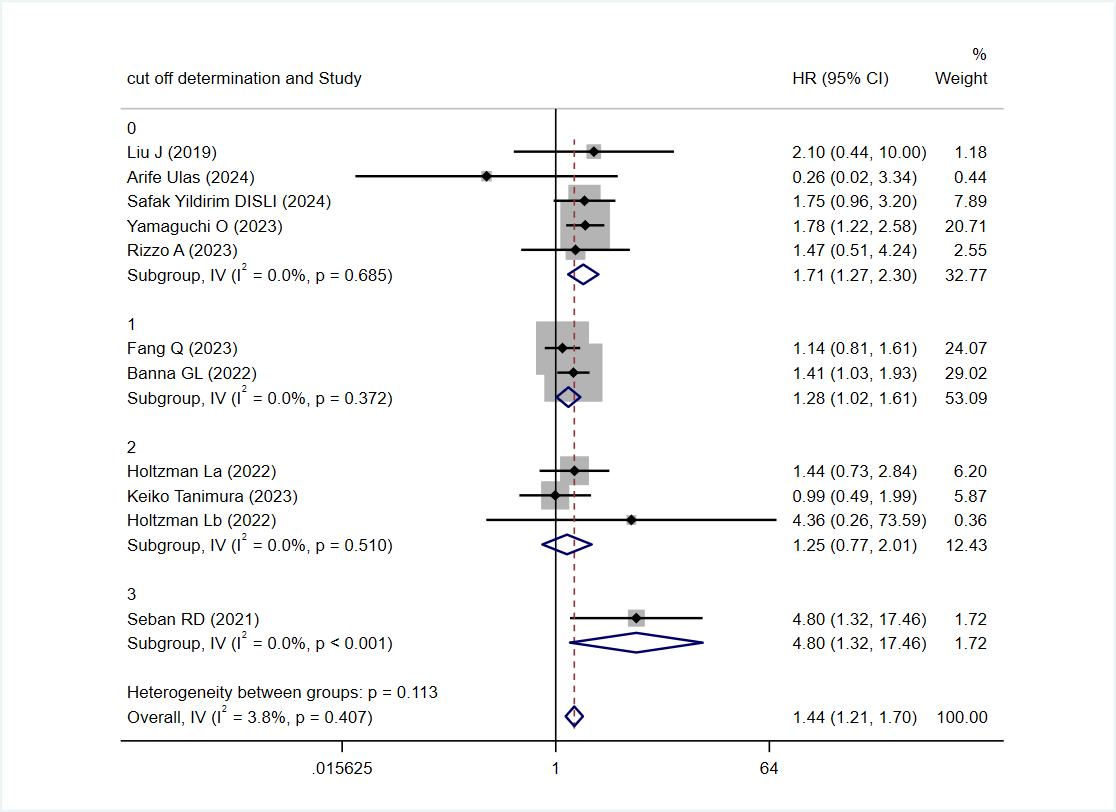

Supplement: Supplementary file 1 [file DataSheet1.zip › supplementary material/Subgroup analyses/OS/Cut-off determination.tif]

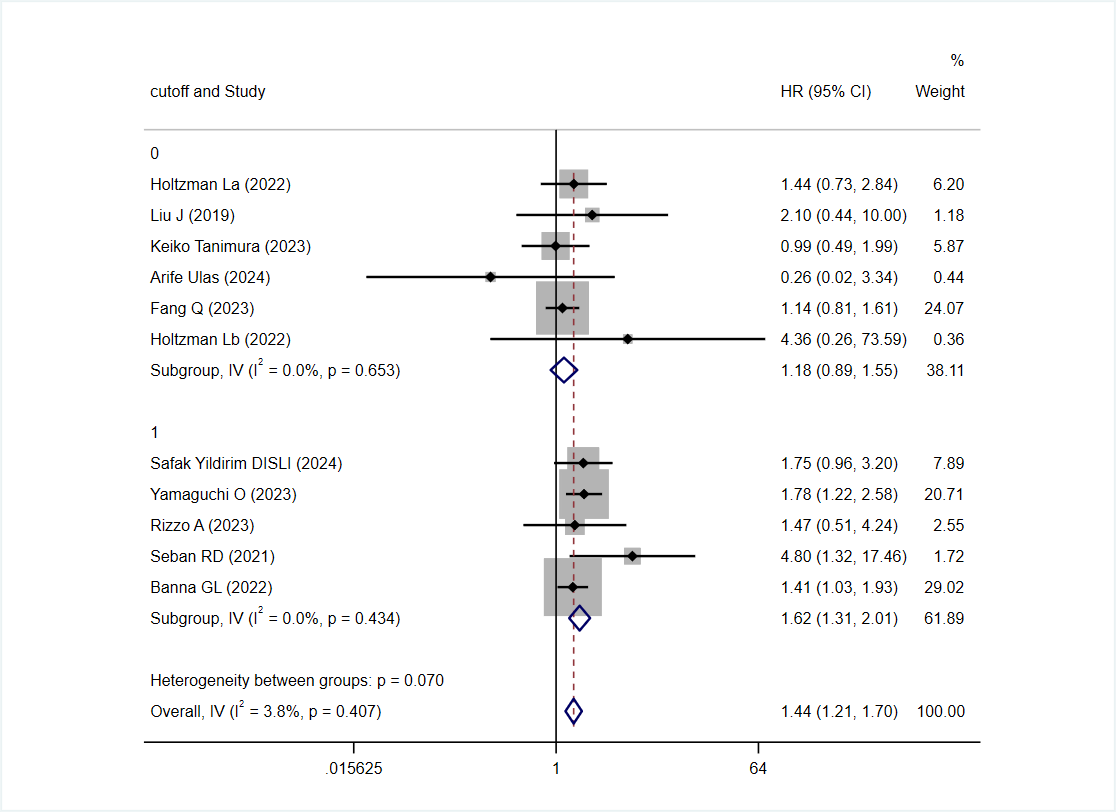

Supplement: Supplementary file 1 [file DataSheet1.zip › supplementary material/Subgroup analyses/OS/Cut-off value of SII.tif]

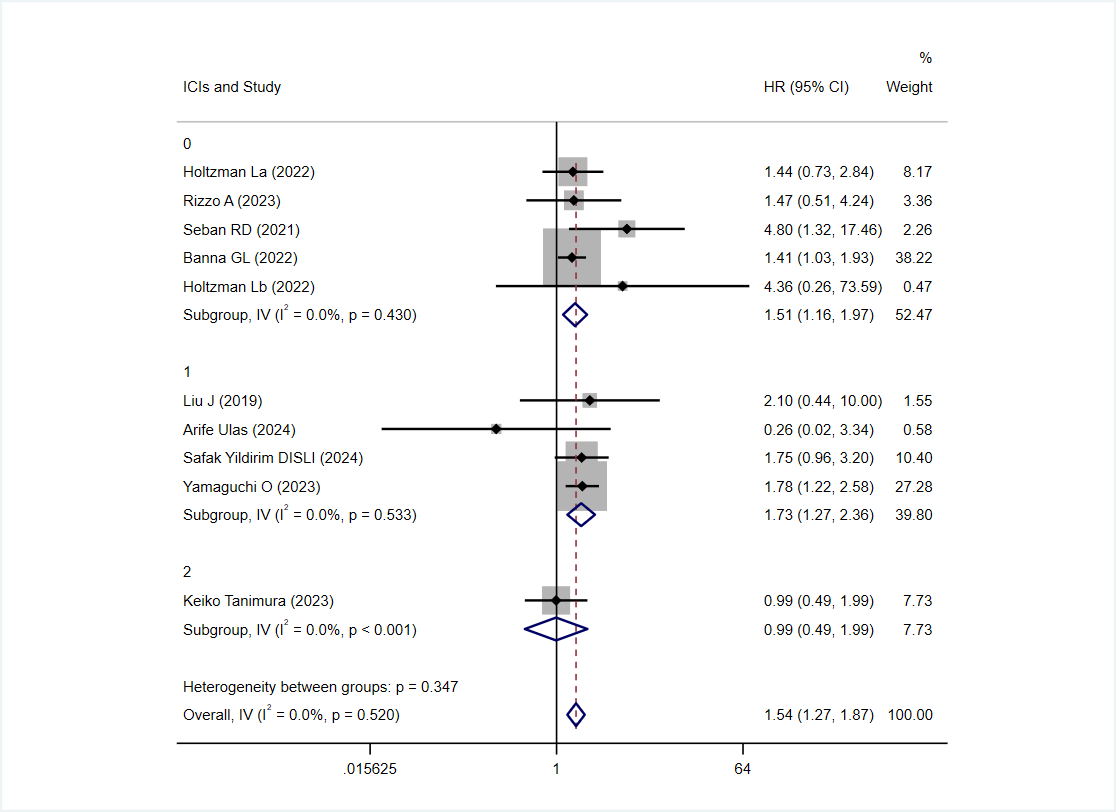

Supplement: Supplementary file 1 [file DataSheet1.zip › supplementary material/Subgroup analyses/OS/PD-(L)1 agent.tif]

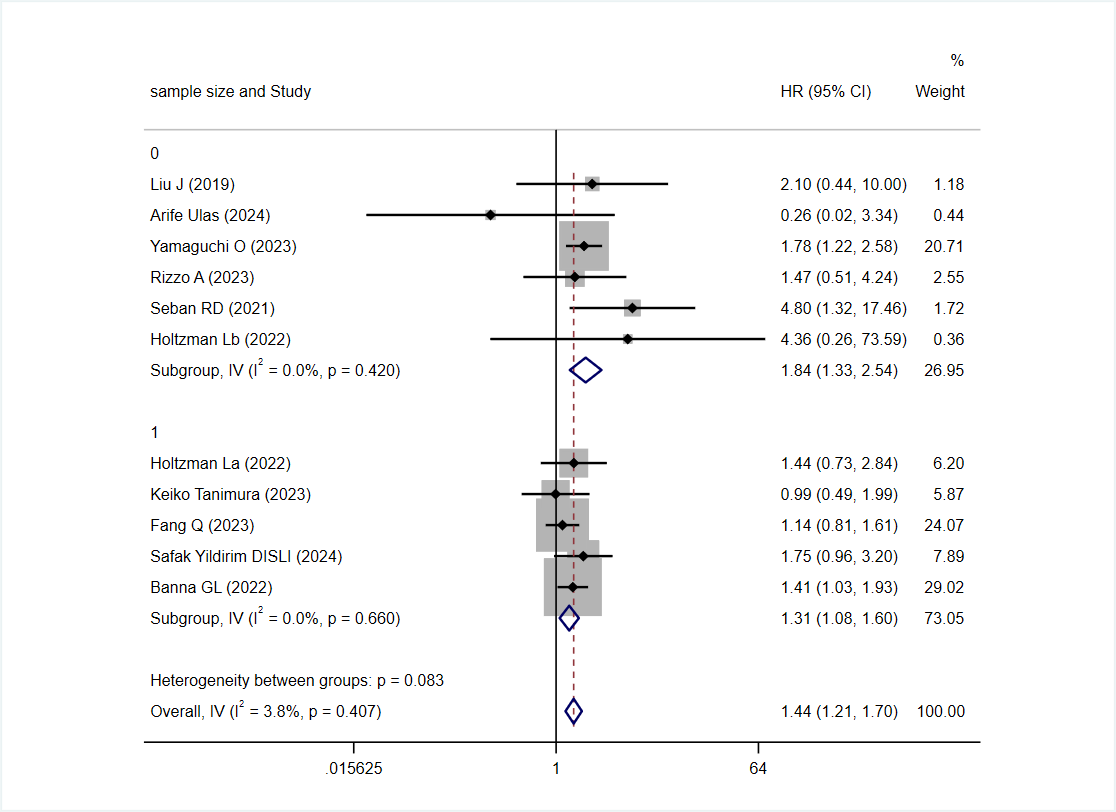

Supplement: Supplementary file 1 [file DataSheet1.zip › supplementary material/Subgroup analyses/OS/Sample size.tif]

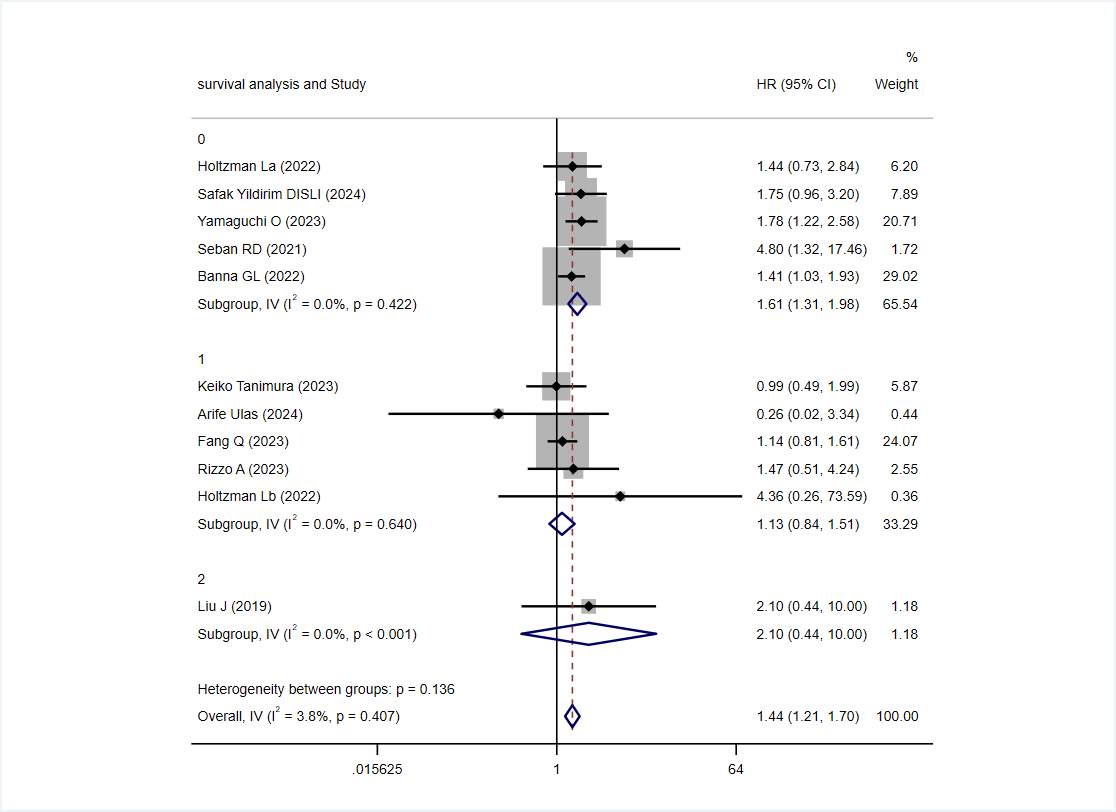

Supplement: Supplementary file 1 [file DataSheet1.zip › supplementary material/Subgroup analyses/OS/survival analysis.tif]

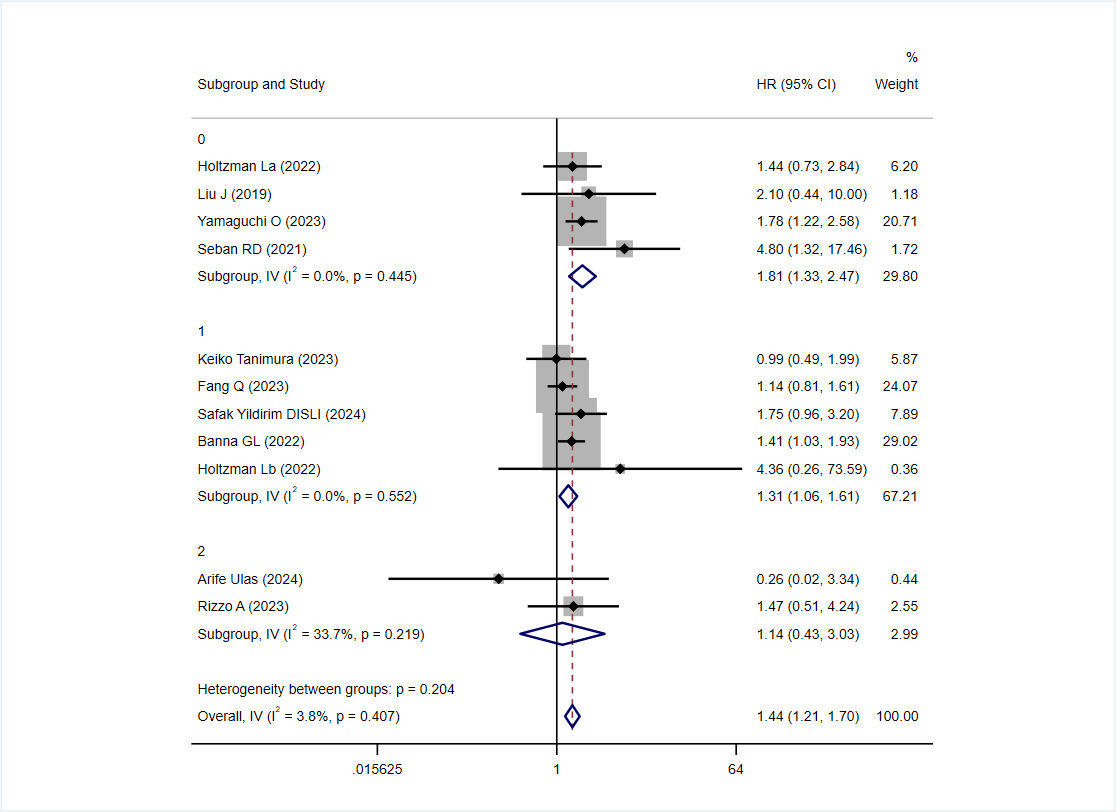

Supplement: Supplementary file 1 [file DataSheet1.zip › supplementary material/Subgroup analyses/OS/treatment.tif]

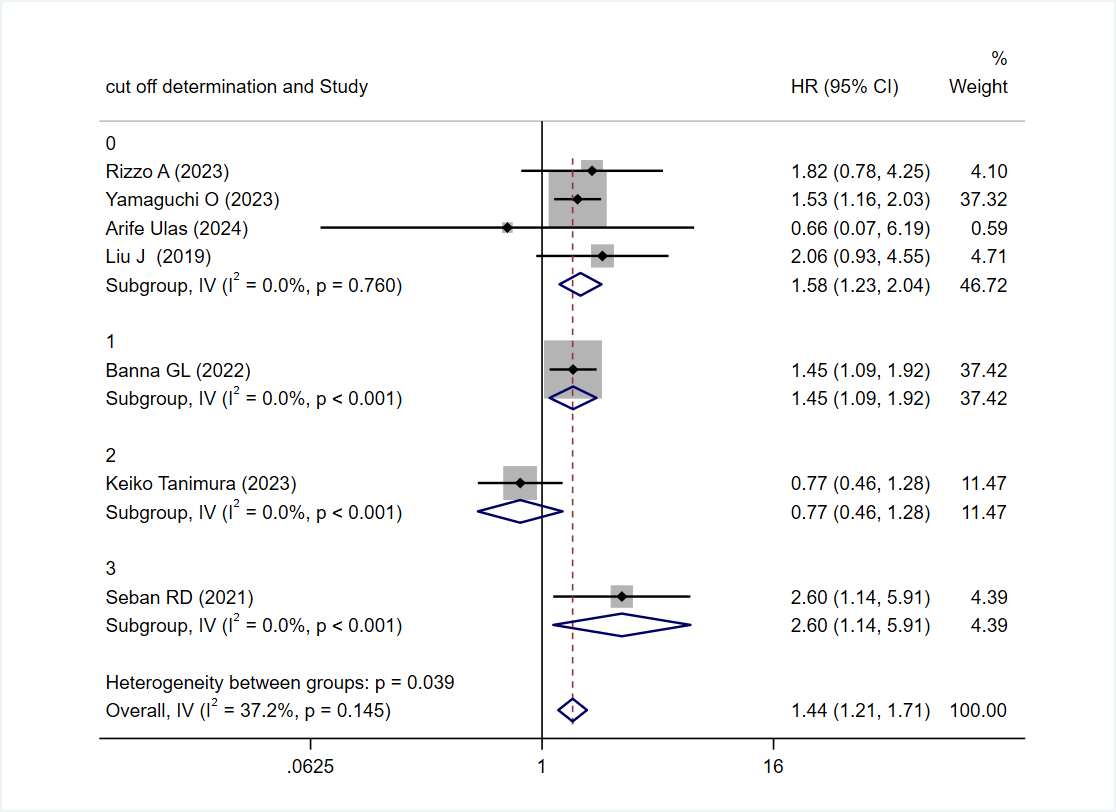

Supplement: Supplementary file 1 [file DataSheet1.zip › supplementary material/Subgroup analyses/PFS/Cut-off determination.tif]

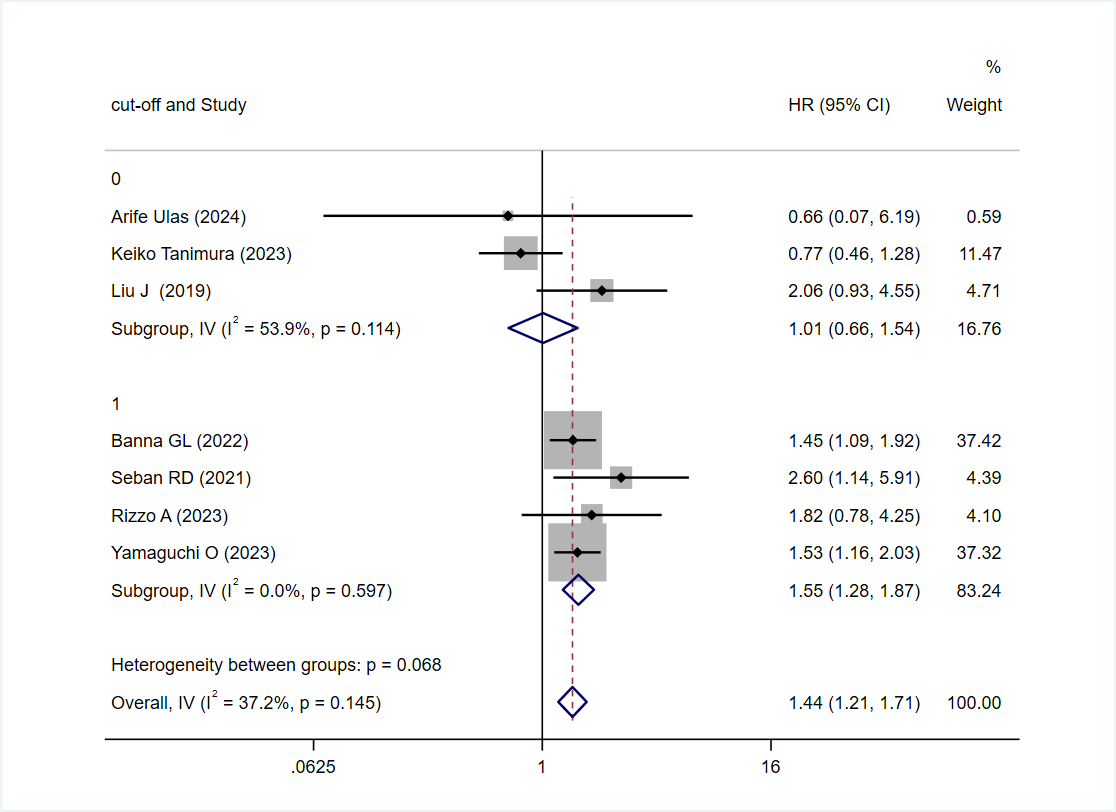

Supplement: Supplementary file 1 [file DataSheet1.zip › supplementary material/Subgroup analyses/PFS/Cut-off value of SII.tif]

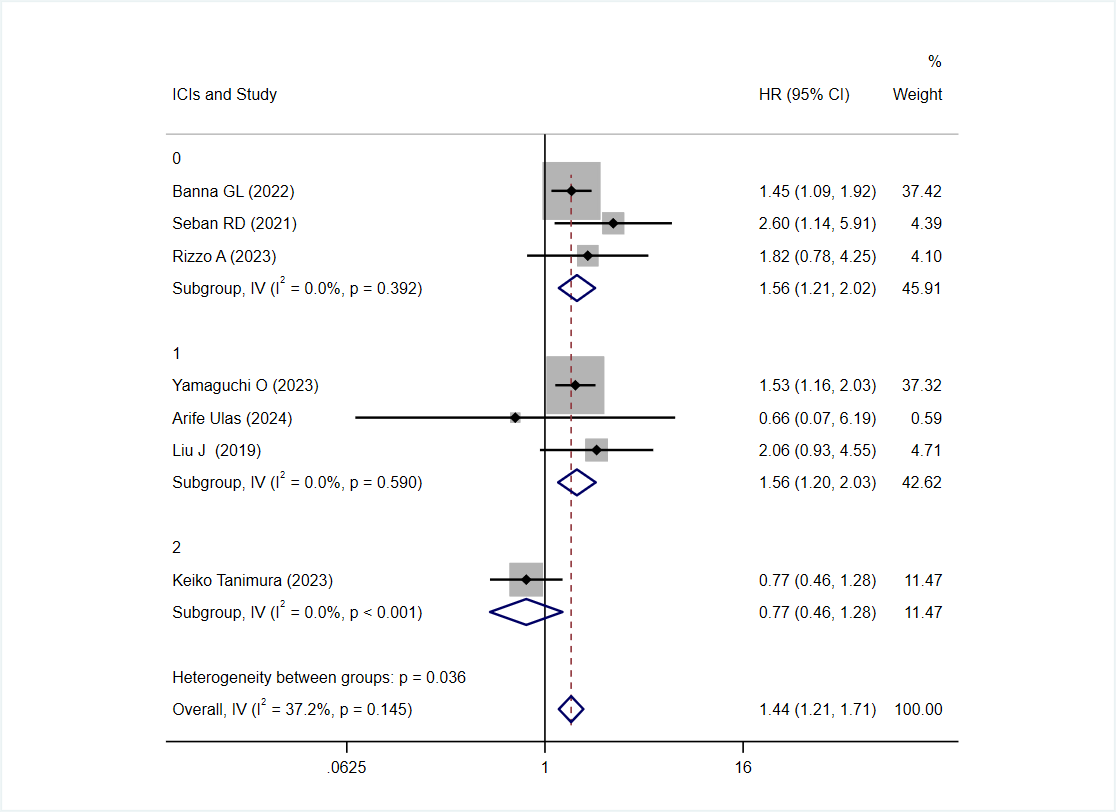

Supplement: Supplementary file 1 [file DataSheet1.zip › supplementary material/Subgroup analyses/PFS/PD-(L)1 agent.tif]

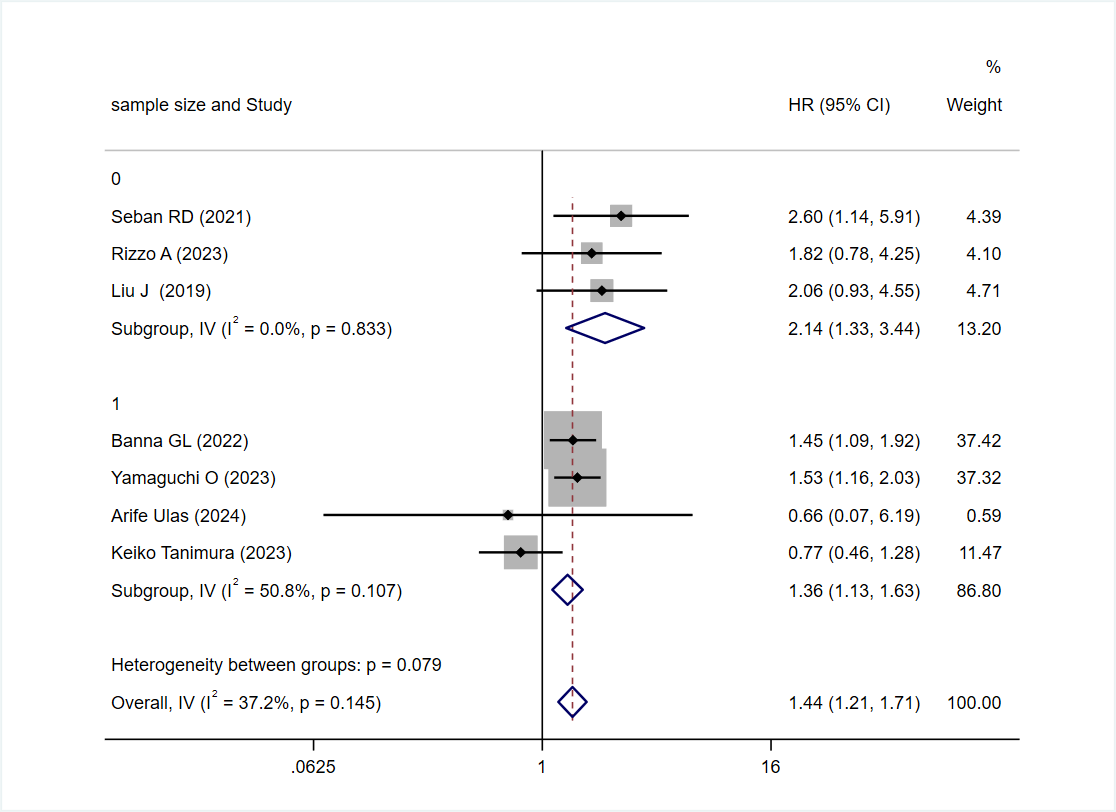

Supplement: Supplementary file 1 [file DataSheet1.zip › supplementary material/Subgroup analyses/PFS/Sample size.tif]

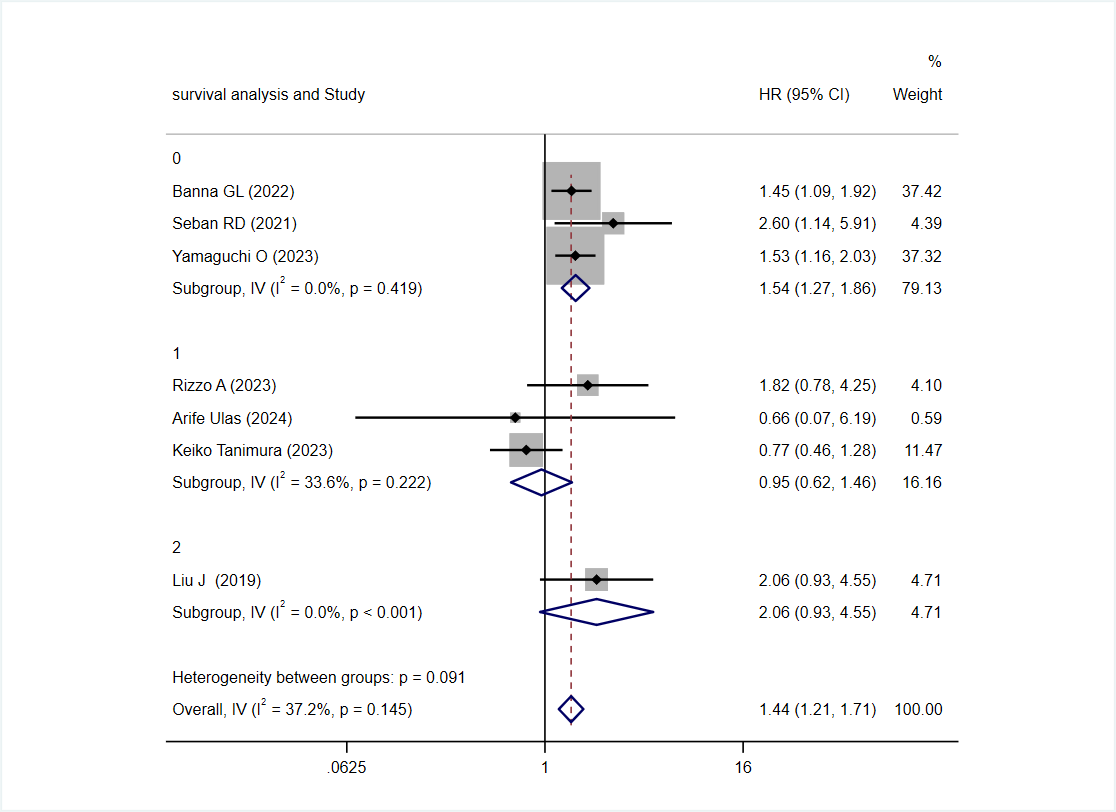

Supplement: Supplementary file 1 [file DataSheet1.zip › supplementary material/Subgroup analyses/PFS/survival analysis.tif]

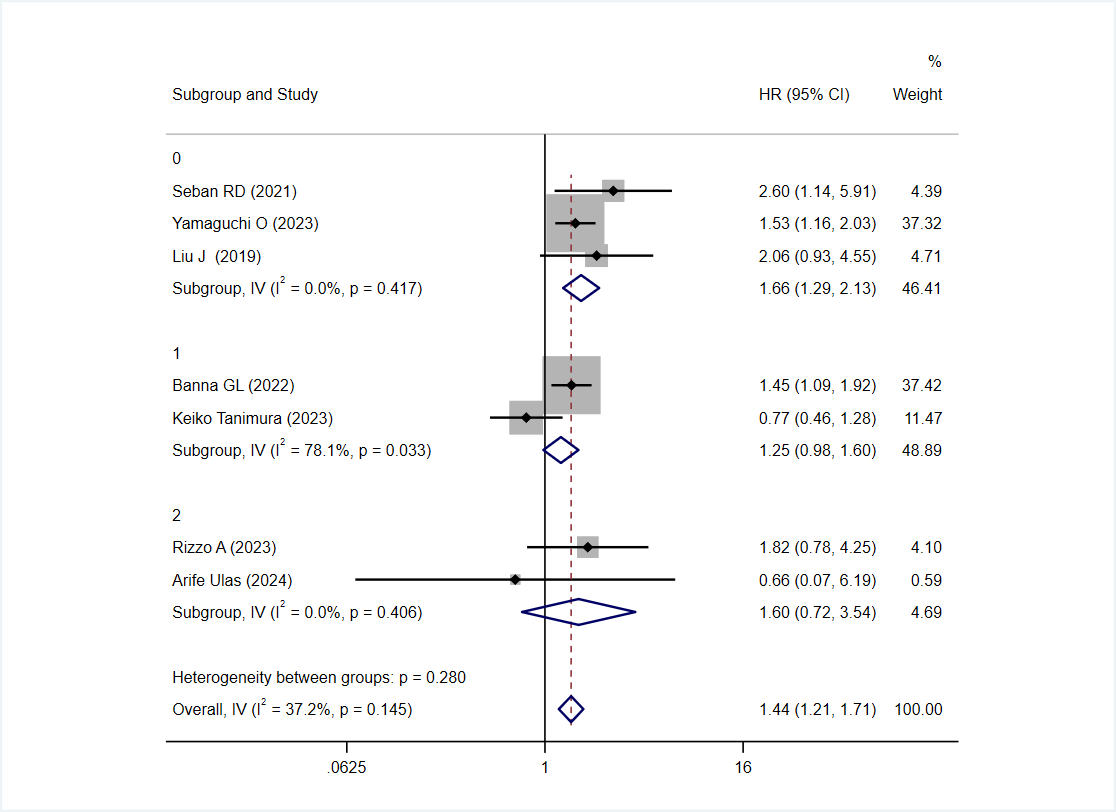

Supplement: Supplementary file 1 [file DataSheet1.zip › supplementary material/Subgroup analyses/PFS/treatment.tif]
